# Qualitative exploration of the modifiable factors for medication adherence among subsidised and self-paying patients in Malaysia

**DOI:** 10.1186/s12913-018-3417-y

**Published:** 2018-08-06

**Authors:** Hamiza Aziz, Ernieda Hatah, Mohd Makmor-Bakry, Farida Islahudin, Najwa Ahmad Hamdi, Ivy Mok Pok Wan

**Affiliations:** 1 Pharmacy Department, Putrajaya Hospital, Federal Government Administrative Centre, Precinct 7, 62250 Putrajaya, Malaysia; 2 Faculty of Pharmacy, Universiti Kebangsaan Malaysia, Jalan Raja Muda Abdul Aziz, 50300 Kuala Lumpur, Malaysia; 3 Family Health Development Division, Ministry of Health, Parcel E, Federal Government Administrative Centre, 62590 Putrajaya, Malaysia; 4 Pharmacy Department, Universiti Kebangsaan Malaysia Medical Centre, Jalan Yaacob Latif, Bandar Tun Razak, Cheras, 56000 Kuala Lumpur, Malaysia

**Keywords:** Medication adherence, Subsidised medication, Self-paying patients, Medication cost

## Abstract

**Background:**

Numerous studies have evaluated the related factors of medication adherence among patients with chronic disease. However, the factors influencing medication adherence and non-adherence among subsidised patients with chronic diseases—for whom medication costs may not be a constraint—remain unexplored. Thus, this study aims to identify and compare the potential factors that may influence subsidised and non-subsidised (i.e., self-paying) patients’ adherence to medication.

**Methods:**

Subsidised and self-paying patients were identified at public and private healthcare institutions in three states of Malaysia. Patients were then purposively selected for semi-structured, face-to-face interviews according to their medication adherence status (including adherent and non-adherent patients), which was measured using the Medication Event Monitoring System (MEMS). Adherence was defined as having 80% or more for the percentage of days in which the dose regimen was executed as prescribed. The interview was conducted from January to August 2016 and during the interviews, patients were asked to provide reasons for their medication adherence or non-adherence. The patient interviews were audio recorded and transcribed verbatim. Data were analysed using thematic analysis with NVivo 11 software.

**Results:**

Thirteen subsidised and 12 self-paying patients were interviewed. The themes found among subsidised and self-paying patients were similar. The factors that influenced adherence to medication include the *‘perceived importance of quality of life’* and *‘perceived benefit or value of the medications’.* A unique factor reported by patients in this study included ‘*perceived value of the money spent on medications’*; more specifically, patients adhered to their medications because they valued the money spent to buy/receive the medications.

**Conclusion:**

Medication adherence among subsidised and self-paying patients was influenced by many factors, including a unique factor relating to their perceptions of the value of money spent on medications.

## Background

Socioeconomic factors such as medication costs are increasingly recognized as important for patients’ health and health-related behaviour [1]. Medication costs may increase patients’ tendency to become non-adherent to their medication [2, 3]. A study done in Turkey on hypertensive patients showed that half of the non-adherent patients failed to take their medication as prescribed because of the high cost of the medications [4]. Besides medication costs, drug benefit caps and higher co-payments were also reported as reasons for medication non-adherence [5, 6]. A systematic review of the influence of patients’ payment scheme on medication adherence showed that a reduction in patient cost-sharing—such as lower co-payment and higher drug coverage and prescription caps—were associated with better medication adherence [7]. Patients who reported that they were non-adherent (including skipping doses, stopping taking a prescription drug altogether, or failing to fill a new prescription) due to medication costs did so because they either lacked the money or wanted to save money [8, 9]. Salas et al., in their systematic review, suggested that a reduction in patients’ pharmacy bills can increase patients’ adherence to their medication [10].

However, a reduction in patients’ cost sharing has shown varied findings with regard to medication adherence. In some studies, medication adherence improved following a reduction in patients’ co-payment [11–13]. For example, adherence to statin increased by about 2.8% when patients’ co-payment was reduced to $0.60 per month [13]. Nevertheless, several studies have shown that medication adherence is rather poor even among patients without medication cost constraints. In a study of diabetes patients in Texas who visited grocery store chain pharmacies or a community clinic for the underserved, over half (56%) were non-adherent to their medication [14]. In another study in the United States, patients with an overactive bladder who received subsidised medications had a similarly low adherence rate, at only 34% [15]; this rate, notably, did not differ significantly from those in previous studies for patients with a co-payment scheme (30.3–53%) [15]. The above-mentioned systematic study further confirmed that medication adherence among patients with medication subsidies (i.e., the cost of medication was minimal or free) was similar to that of patients with other payment schemes [7]. Although there are numerous studies on the medication adherence rate among patients with medication subsidies, the reasons for the non-adherence in this population remains unexplored. It also remains unclear whether these reasons differ from those among self-paying patients.

In Malaysia, medical care is heavily subsidised by the public healthcare system. All Malaysian citizens, regardless of their income, have access to medical care at government hospitals and clinics, wherein they need to pay a minimum of Ringgit Malaysia (RM) 1 to RM5 (USD 0.25 to 1.24) per visit. The cost for a visit includes a medical consultation, laboratory investigation, and medications. Members of the public can also choose to seek treatment in private health care settings, which are usually funded out-of-pocket or by third-party insurers. A consultation fee for a general practitioner’s visit in a private healthcare setting is currently capped at RM30 to RM120 (USD 7.40 to 29.60) per visit. This does not include the costs for the required laboratory examinations, procedures, or medications. Currently, the government has no control over medication prices in private healthcare settings; thus, the difference in medication costs between public and private settings might be high. Despite the heavy subsidies for medication in the public health care system, the findings of a study on returned unused medication at a public hospital in Malaysia were worrisome: that is, they found that the average cost of the medication returned to the pharmacy counter was rather high, at around RM42.35 (USD 10.50) per patient [16]. If this was similar in public healthcare institutions across Malaysia, it would lead to millions of ringgit in losses for the government per year [16]. One of the possible reasons for the returned unused medication is medication non-adherence. Similarly, another study of hypertensive patients seeking treatment in public health care institutions showed that only 50% adhered to their medication [17]. Medication non-adherence may lead to wastage of government subsidies and increase in chronic disease complications. Since burden of chronic diseases is rapidly increasing which had contributed to 63% (2008) and 73% (2014) of reported death worldwide and in Malaysia, respectively [18, 19], investigating the factors that influence medication adherence among in patients with chronic disease that with and without medication subsidies will likely help in understanding the medication-taking behaviour of these two groups.

### Objective

This qualitative study aims to identify and compare potential factors that might influence subsidised or non-subsidised (i.e., self-paying) patients’ adherence to their medications.

## Methods

This study was conducted using qualitative semi-structured interviews of patients with medication subsidies and self-paying patients. Patients who received medication subsidies from public hospitals or a health clinic in Kuala Lumpur, Selangor and Negeri Sembilan were deemed the subsidised group. Meanwhile, patients who sought treatment from private healthcare institutions in Kuala Lumpur and Selangor or who were paying for their medication were deemed self-paying patients. Sampling sites were chosen based on convenience and according to the institution’s approval.

Prior to the interviews, the authors evaluated patients’ adherence to their medication using the Medication Event Monitoring System (MEMS) bottle cap, which recorded and stored patients’ dosing events whenever the medication container was opened and closed. Using convenience sampling, patients who agreed to participate were provided with a MEMS bottle. Only one of the respondent’s chronic illness medication, that deem was the most expensive medication, was repacked into the MEMs bottle which were returned after four weeks. Among the subsidised patients, the most expensive medication was identified through screening the medication costs printed on the drug label. In public healthcare institutions, the medication label contains the cost of the medication to inform patients of how much of the medication cost is subsidised by the government. By contrast, among the self-paying patients, the most expensive medication was identified through itemized billing or by using patients’ self-reported costs. Adherence was defined as having 80% or more for the percentage of days in which dose regimen was executed as prescribed. This was confirmed by patients during their invitation into the study and also confirmed through pill count assessment during the interview. Using the information obtained from this study, purposive sampling was performed to select adherent or non-adherent patients from among the subsidised and self-paying groups.

Upon their agreement to participate, face-to-face interviews were conducted at patients’ homes by HA. Interviews were conducted in Malay or English, depending on patients’ preference, and each interview lasted for approximately 30–40 min. With patients’ consent, the interviews were tape-recorded. The interview was conducted from January to August 2016 and during the interview, patients were asked about the importance of medication, the reasons for medication adherence and non-adherence, and the cost estimation for the medication received. A pilot interview using this topic guide was conducted with three patients and an academician who was an expert in qualitative studies. The input from the pilot interviews was used to refine the topic guide. The topic guide used for the interviews is shown in Appendix.

The interviews were transcribed verbatim by HA and checked by EH. To reduce the risk of biases in the analysis, any information that could be used to identify patient’ groups (subsidised and self-paying patients) were removed from the transcripts. Patients were recruited until saturation was achieved—that is, until no new themes appeared in two consecutive interviews.

The interview data were then analysed using the framework analysis approach with NVivo 11 software. Commonly reported factors relating to medication adherence and non-adherence, such as perceived susceptibility, severity, benefits, barriers, cues to action, and self-efficacy (all of which are proposed in the Health Belief Model (HBM) of Medication Adherence), were used to classify patients’ responses [20]. The HBM which is a psychological model that explains and predicts health behaviours was chosen to underpin the framework analysis in the current study because it is widely use and well-tested [21]. Studies have reported that HBM explained medication adherence well with percentage of reported variance in patients with different diseases of between 26 and 88% [22]. In addition, a systematic review and meta-analysis study of theory use in medication adherence intervention research found that the intervention using HBM approaches had the largest effect sizes (0.477) compare to other theory-based interventions [23]. This may reflect at the moment HBM may be the best model that able to predict, explain and use for intervention for patients’ medication adherence behaviour.

A factor was considered unique to this study if it was not previously reported to cause medication adherence/non-adherence according to the HBM of medication adherence. Identified factors were cross-checked by EH and any disagreement related to the factors was discussed with a research group member until consensus was achieved. The identified factors relating to medication adherence and non-adherence were then compared between the groups. All of the Malay quotations were translated into English using forward and backward translation by EH. The translated quotes were re-read and listened to on the audio records by the research team to ensure that the essence of the information was retained in the translation. The study was approved by the Research Ethics Committee of Universiti Kebangsaan Malaysia (UKM 1.5.3.5/244/ NF-056-14) and the Medical Research and Ethics Committee of Ministry of Health Malaysia (NMRR-14-1255-22,473).

## Results

Saturation of themes related to the factors that might influence adherence and non-adherence to medications was achieved upon recruitment of 25 patients, including 13 in the subsidised group and 12 in the self-paying group. Participants’ demographic and characteristics are presented in Table 1. The mean age (standard deviation [SD]) of the patients was 52.8 (11.26). The gender ratio was relatively equal, with 11 of the patients being male and 14 female. Patients had between two and four illnesses, including diabetes mellitus, hypertension, heart disease, and hyperlipidaemia. The majority of the patients received three medications for their diseases (range: 3–9). There was no significant difference between adherent and non-adherent patients’ demographics or other characteristics, such as education level and number of medications (*P* > 0.05).

**Table 1:** Patients’ demographic and characteristics

Description	Total n (%)/mean (±SD) (*n* = 25)	n/mean (±SD/%)	
		Subsidised (*n* = 13)	Self-paying (*n* = 12)
Age	52.84 (11.26)	50.62 (12.63)	55.91 (7.98)
Gender			
Male	11 (44)	6	5
Female	14 (56)	7	7
Monthly income			
< RM1000	5 (20)	1	4
RM1001–RM3000	9 (36)	8	1
> RM3001	11 (44)	4	7
Highest educational level			
Primary school	6 (24)	5	1
Secondary school	6 (24)	4	2
College/university	13 (52)	4	9
Marital status			
Single	1 (4)	1	0
Married	24 (96)	12	12
Medication adherence status^a^			
Adherence	13	8	5
Non-adherence	12	5	7
Attended medication counselling			
Yes	12 (48)	5	7
No	13 (52)	8	5
Number of health problems	3.04 (±0.68)	3 (0.68)	2.75 (0.6)
Number of medications	5.52 (±1.87)	6.15 (1.96)	4.67 (1.43)

^a^Measured using Medication Event Monitoring System (MEMS)

### Common factors for medication adherence and non-adherence

Several factors influenced patients’ adherence to medication in both the subsidised and self-paying groups. These included *motivation*, such as *perceived importance of quality of life* and *perceived value of medication*; *cues to action*; *trustworthiness of healthcare recommendations*; and *positive social support*. There were, however, slight differences in the *motivation* theme between the subsidised and self-paying groups, so the themes will be explained in further detail in the next section. The common reasons for medication adherence can be illustrated in the following example quotations:

*‘My parents always asked me to continue my follow-ups with the doctor because they don’t want me to be blind or to have kidney complications like those experienced by one of our family members’* (Subsidised patient (SP) 3) [Cues to action].

*‘Every morning my husband used to give me my medications. When it is time to take the medication, my husband will remind me to do so and would give me my medication. My husband also helped me to inject insulin’* (SP 11) [Positive social support].

*‘I trust the doctors who give the prescriptions’* (Paying patient (PP) 2) [Trustworthiness of healthcare providers]*.*

The common reasons for non-adherence were *perceived barriers to medication adherence,* such as longer duration of taking the medication, feeling unpleasant as a result of the treatment (e.g., feeling bored or experiencing drug side effects), and forgetfulness, and *perceived non-severity of the disease.* The latter reason might be due to a lack of knowledge of the disease or treatment. Notably, there were no differences in reasons for medication non-adherence between the subsidised and self-paying patients, and none of the patients reported medication cost as a factor for non-adherence.

*‘Sometimes I feel too bored to take my medication, as there are too many. (Also) I felt like vomiting (when taking it)’* (SP 2) [Perceived barriers to medication adherence].

*‘Sometimes I do not inject my insulin, especially when I go back to my hometown…I unintentionally forget to bring my medications along’* (SP 6) [Perceived barriers to medication adherence]*.*

*‘When I feel better, I do not take my medications’* (SP 10) [Perceived non-severity of the disease]*.*

*‘My specialist asked me to take my heart medication three times a day. I can take the medication accordingly but I am not sure whether my body can tolerate the thrice daily dosing? Thus, I have reduced the dosage on my own and take it twice a day’* (SP 8*)*[Perceived non-severity of the disease]*.*

### Motivation

In both the subsidised and self-paying groups, patients who adhered to their medication tended to be motivated to take their medication as prescribed. However, the reasons for this motivation differed between the groups. Patients with medication subsidies tended to feel motivated to take their medication because they *perceived the importance of quality of life*; in other words, they wanted to stay healthy or avoid disease complications and reduce their symptoms. This was also found to be the source of motivation for self-paying patients.

*‘I take the medication as part of an effort (to be healthy) because I know that I have a disease. God-willing, I want to have better health’* (SP12)*.*

*‘I fear the disease complications that might occur if I do not take my medication. I am afraid of being paralyzed or falling down’ (SP1).*

*‘I take medication so that I am healthy. If I am healthy, I can feed myself, walk, and go to the toilet independently without any problems’* (PP1)*.*

*‘When I’m healthy, I can join in local community activities. I can go to religious activities, or go to the Al-Quran class. But if I’m not well, I have to stay at home. I can’t meet with others. It sure is boring for me who used to work, right?’ (PP 7).*

However, for self-paying patients, the motivation for medication adherence was *the perceived value of the medication,* in term of the benefit related to the prevention of incurred costs due to non-adherence. Patients said that they felt that they had to take their medication as prescribed to prevent costs related to non-adherence, such as increases in the number of medications and loss of income following disease complication.

*‘It is my responsibility to maintain my best possible health. Although I am old, that is not a reason why I shouldn’t take my medicine or care about my health. I want to live happily with my family. If I were sick, I may trouble others or make their life difficult, and also will cause an increase in medication costs’* (PP 5)*.*

*‘If I were sick, how can I find* money*? I may just lie at home (doing nothing), right? That is why I take my medications, although it quite expensive’* (PP 4)*.*

### Perceived value of money spent

A unique theme for medication adherence was found in both the subsidised and self-paying patient groups: that is, they were adherent to their medication because they valued the money that they spent on it. Subsidised patients felt that tax-payers’ money would be wasted if they did not take the medication as prescribed, while self-paying patients adhered because they had spent their own pocket money on buying the medication.

*‘Thank God that I can get my treatment from the government hospital. Some patients cannot afford to get their treatment. Medication is expensive. We who get free medications should appreciate it and be grateful’* (SP13)*.*

*‘If I bought the medications but did not take it, I would be considered stupid…’* (PP 4).

Interestingly, almost all of the subsidised patients underestimated or were unsure of the cost of their medication, despite it being clearly printed on the medication labels. Furthermore, despite their underestimation of the total costs of these medications, patients believed that the medications received from government healthcare institutions were of comparable quality to those administered by private healthcare institutions.

## Discussion

This qualitative study was conducted to explore the potential factors influencing subsidised patients’ adherence to their medication. To investigate these factors, the authors of this study explored subsidised patients’ reasons for taking and not taking their medications and compared them to those of patients who were self-paying for their medications. The authors found that *motivation*—in particular, *the perceived importance of quality of life and perceived value of the medication*—was a reason for adherence in both the subsidised and self-paying patient groups. Motivation is an important element for medication adherence likely because it influences behaviour change [24]. Motivation can be defined in the psychology literature as ‘the psychological forces or energies that impel a person towards a specific goal’ [25]. The term ‘motivation’ is often used to refer to reasons for an action (e.g., ‘what is your motive?’) as well as one’s level of interest in that action (e.g., ‘how motivated are you?’). In this way, motivation can be determined by assessing patients’ readiness and willingness to change [25]. When patients perceive that making the change will benefit them and they perceive no or few barriers to making that change, the change will likely take place. By contrast, if the real or perceived barriers to making the change outweigh the positives, the change is unlikely to occur [25].

In the present study, patients who adhered to their medication reported being motivated to do so in order to take care of their health and avoid disease complications (*perceived importance of quality of life)*. This accorded with the findings of Wu et al. [26] for patients with heart failure, who expressed a strong motivation for medication adherence in order to be healthy and have good quality of life. Similar findings were obtained in a review by Casula et al. [27], wherein adherence was influenced by patients’ motivation to manage their disease, sufficient knowledge and beliefs about their illness, and awareness of the consequences of poor adherence to medications.

Interestingly, besides the *perceive importance of quality of life,* self-paying patients were motivated to adhere to their medication as a result of the *perceived value of medication.* In other words, they considered the potential costs incurred as a result of medication non-adherence. Such costs could be financial (e.g., money spent on treating disease complications and a loss of income)*.* ‘Perceived value’ can be defined as a trade-off between perceived benefit and costs, as reported in the HBM [28]. In previous studies, patients’ decision to take medications as prescribed depended on their perceptions of the value of their medications, such as the ratio of benefit of taking or buying the medication to the amount of money spent [29]. In a study by Fuldeore, medication purchasing behaviour, which may promote patients’ adherence to their medication, was influenced by patients’ perceptions of the benefit and value of the medication [28]. In Fuldeore’s study, adherence increased with the perceived value of the medication [28]. Similar findings were reported by Wong et al. [29], who conducted a study on the association between increased co-payment and medication adherence between White and Black veterans; they found that the perceived value of medication was related to the willingness to pay more for their medication, and was one of the contributing factors to medication adherence [29].

Economic behaviour researchers have shown that money is a commonly used, and important, means of motivating behaviour change [30]. For example, financial incentives tend to be offered to employers to boost their motivation to achieve the institution’s target goals. In the current study, self-paying patients who adhere to their medication might be motivated to be adherent as they valued their medication because it was important for maintaining their health, as well as allowing them to work and earn money to support themselves and their families. Additionally, since patients were self-paying for their medical and medication bills, they become more aware of the significance of healthcare expenditures related to disease complications or increases in the number of medications prescribed. This made them aware of the value of their current medication, which in turn motivated them to take care of their health and adhere to their medication.

Another important theme found for medication adherence was *‘perceived value of the money spent’.* In the current study, patients who were adherent to their medication valued it because of the *money spent to purchase the medication*. Patients felt that because they were using either their own or tax-payer money, they should not waste it and therefore should utilize the medication properly. Money has been found to have physiological value to people. Lea and Webley [31], in their study, found that money has value and emotional charge independent of its economic use. For example, establishing a penalty for breaking rules has been found to relieve people from their moral obligation to act appropriately. Money’s persuasive power depends on how it affects people’s cognition [32]. In Shiv et al.’s study, money was found to be a cue of physical effects—that is, participants who were given a drink that purportedly helped their mental acuity reported that their performance in solving puzzles was significantly lower when they received a discounted drink compare to when they received a regular-priced drink [33]. The authors claimed that cost might have some value or quality that can influence people’s behaviour differently. This was similar to how subsidised patients in the current study perceived the total cost of the medication received. Almost all subsidised patients underestimated the cost of medications, despite it being clearly printed on the medication’s label. Thus, subsidised patients might value their medication less because they received it at minimal or no cost.

None of the participants in either group reported medication cost as a reason for non-adherence. Patients in subsidised groups have, in previous studies, reported that multiple factors affect medication non-adherence, such as forgetfulness, pill burden and regimen complexity, the medication being ineffective, and a lack of knowledge regarding the disease and its treatment [15, 25, 34–36]. Similar factors of medication non-adherence were reported by self-paying patients. Medication cost was not likely a factor because subsidised patients did not need to pay for their medication, whereas self-paying patients, at least in the present study, could afford to purchase their medications.

The limitation of the current study is that it might have only included patients who could afford to purchase their medications. Since self-paying patients were recruited from pharmacy counters, the authors might have missed patients who did not turn up for refills because they could not afford to purchase the refills. However, finding such patients might be difficult because the government of Malaysia provides subsidised health services to all Malaysians, regardless of their socioeconomic background. Thus, patients who cannot afford to purchase their medication in private healthcare settings will usually use subsidised healthcare services from a Ministry of Health institution.

## Conclusion

Medication adherence among subsidised patients was influenced by many factors, including patients’ perceptions of the importance of their quality of life. A unique factor for medication adherence among self-paying patients was the perceived value of the medication, which was related to the impact of medication non-adherence on patients’ finances. Medication adherence was also influenced by the perceived value of money.

## Appendix

### Interview topic guide

1. Patients’ perceptions of their health condition.
2. Patients’ view of their medication adherence status.
3. Reasons for medication adherence/non-adherence.
4. Patients’ perceptions and experience of the quality of medication therapy.
5. Perceived cost estimation for subsidised medication received.

## Abbreviation

EH: Ernieda Hatah
HA: Hamiza Aziz
HBM: Health Belief Model
MEMS: Medication Event Monitoring System
PP: Paying patient
RM: Ringgit Malaysia
SP: Subsidised patient
USD: United States Dollar
